# Fulvic Acid Attenuates Resistin-Induced Adhesion of HCT-116 Colorectal Cancer Cells to Endothelial Cells

**DOI:** 10.3390/ijms161226174

**Published:** 2015-12-09

**Authors:** Wen-Shih Huang, Jen-Tsung Yang, Chien-Chang Lu, Shun-Fu Chang, Cheng-Nan Chen, Yu-Ping Su, Ko-Chao Lee

**Affiliations:** 1 Graduate Institute of Clinical Medical Sciences, College of Medicine, Chang Gung University, Taoyuan 333, Taiwan; wen1204@adm.cgmh.org.tw (W.-S.H.); cclu999@gmail.com (C.-C.L.); 2 Division of Colon and Rectal Surgery, Department of Surgery, Chang Gung Memorial Hospital, Chiayi 613, Taiwan; 3 Department of Neurosurgery, Chang Gung Memorial Hospital at Chiayi, Chang-Gung University College of Medicine, Chiayi 613, Taiwan; jents716@ms32.hinet.net; 4 Division of Colorectal Surgery, Department of Surgery, Chang Gung Memorial Hospital-Kaohsiung Medical Center, Chang Gung University College of Medicine, Kaohsiung 833, Taiwan; 5 Department of Medical Research and Development, Chang Gung Memorial Hospital Chiayi Branch, Chiayi 613, Taiwan; sfchang@cgmh.org.tw; 6 Department of Biochemical Science and Technology, National Chiayi University, Chiayi 600, Taiwan; cnchen@mail.ncyu.edu.tw; 7 Department of Orthopaedics and Traumatology, Taipei Veterans General Hospital & School of Medicine, National Yang-Ming University, Taipei 112, Taiwan; ypsu@vghtpe.gov.tw

**Keywords:** colorectal carcinoma, fulvic acid, intercellular adhesion molecule-1, resistin, vascular cell adhesion molecule-1

## Abstract

A high level of serum resistin has recently been found in patients with a number of cancers, including colorectal cancer (CRC). Hence, resistin may play a role in CRC development. Fulvic acid (FA), a class of humic substances, possesses pharmacological properties. However, the effect of FA on cancer pathophysiology remains unclear. The aim of this study was to investigate the effect of resistin on the endothelial adhesion of CRC and to determine whether FA elicits an antagonistic mechanism to neutralize this resistin effect. Human HCT-116 (p53-negative) and SW-48 (p53-positive) CRC cells and human umbilical vein endothelial cells (HUVECs) were used in the experiments. Treatment of both HCT-116 and SW-48 cells with resistin increases the adhesion of both cells to HUVECs. This result indicated that p53 may not regulate this resistin effect. A mechanistic study in HCT-116 cells further showed that this resistin effect occurs via the activation of NF-κB and the expression of intercellular adhesion molecule-1 (ICAM-1) and vascular cell adhesion molecule-1 (VCAM-1). Co-treating cells with both FA and resistin revealed that FA significantly attenuated the resistin-increased NF-κB activation and ICAM-1/VCAM-1 expression and the consequent adhesion of HCT-116 cells to HUVECs. These results demonstrate the role of resistin in promoting HCT-116 cell adhesion to HUVECs and indicate that FA might be a potential candidate for the inhibition of the endothelial adhesion of CRC in response to resistin.

## 1. Introduction

Fulvic acid (FA) is a natural, acidic organic polymer extracted from humus found in soil, sediment, or aquatic environments [1,2]. It is composed of a mixture of closely related complex aromatic polymers, and spectroscopic and chemical analysis have demonstrated the presence of multiple active functional groups, including aromatic rings and phenolic hydroxyl, ketone carbonyl, quinone carbonyl, carboxyl and alkoxyl groups [3]. Traditionally, FA is used as a supplement, as these active functional groups help the absorption of other essential nutrients into the body [1]. Recently, it was reported that FA has antioxidant properties and apparent neuro-protective effects [4]. Additional antimicrobial and anti-inflammatory properties of FA have also been reported [5,6]. These results from pharmacological studies of FA imply that FA may modulate and control the pathophysiology of multiple diseases. However, the detailed roles and mechanisms underlying these regulatory effects of FA remain unclear.

Colorectal cancer (CRC) is one of the leading causes of malignancy deaths worldwide [7]. Its incidence rate has accelerated in developing countries over the past few decades [8]. Recently, accumulating studies have shown that central obesity may be the most important risk factor for CRC development [9]. Moreover, obesity (body mass index, BMI > 25 kg/m^2^) increases CRC risk 7%–60% compared to an individual with BMI < 25 kg/m^2^ [10,11]. Adipose tissue has been revealed as an endocrine organ, releasing various adipokines with multiple regulatory activities for the pathogenesis of disease [12,13]. Resistin is a newly discovered adipokine. Recent studies have indicated that the plasma levels of resistin are increased in many inflammation-related disorders such as atherosclerosis and arthritis [14]. Increasing evidence has also revealed that serum resistin positively correlates with the development and metastasis of malignancies [15]. Although the studies have some discrepancies, the circulating levels of serum resistin may serve as biomarkers for the pathogenesis and progression of many cancers, in particular CRC. However, information is lacking about the detailed regulatory mechanisms of resistin in CRC development.

Cancer metastasis is the most life-threatening clinical issue associated with human cancer. It results in poor prognosis and is responsible for approximately 90% of patient mortality [16,17,18]. Metastasis is facilitated by the adhesion of cancer cells to the endothelium in the second tissue site. Moreover, cell adhesion molecules (CAMs) including selectins, integrins, and immunoglobulin superfamily proteins play critical and necessary roles in metastasis [19]. The intercellular adhesion molecule-1 (ICAM-1) and vascular cell adhesion molecule-1 (VCAM-1) are two immunoglobulin superfamily adhesion molecules. Both contribute to the endothelium adhesion of cancer cells and the immune responses of cancers [16,20]. The expression of ICAM-1 and VCAM-1, which are regulated by the transcription factor NF-κB, in endothelial cells initiates the adhesion of cancer cells and monocytes to the endothelium [16,21,22]. Moreover, our previous study showed that ICAM-1 and VCAM-1 were also expressed in hepatocellular carcinoma (HCC) and promoted HCC cell adhesion to the endothelium [15]. Hence, ICAM-1 and VCAM-1 in both cancer and endothelial cells may play synergistic roles in metastasis progressions.

In this study, we determined the effects of resistin and FA on the endothelium adhesion of human CRC cells and the underlying mechanism. We found that resistin activates NF-κB to induce ICAM-1 and VCAM-1 expression in HCT-116 cells, promoting CRC cell adhesion to endothelial cells. However, FA attenuated these resistin effects on HCT-116 cells. Our data reveal the resistin regulatory effects on CRC adhesion to the endothelium and suggest that FA may serve as a potential therapeutic candidate to inhibit the endothelium adhesion of CRC under resistin stimulation.

## 2. Results

### 2.1. Resistin Induces Adhesion of HCT-116 and SW-48 Cells to HUVECs (Human Umbilical Vein Endothelial Cells)

**Figure 1:**
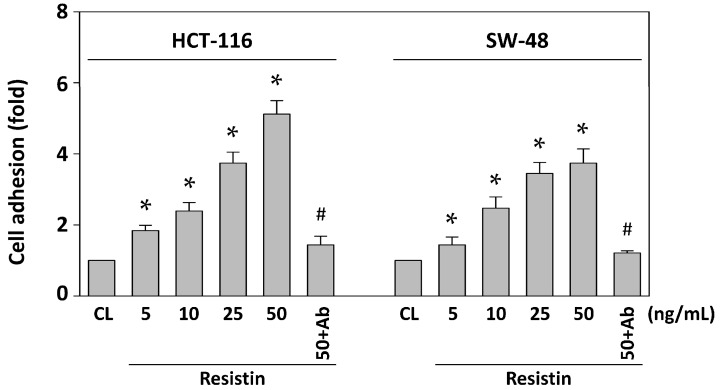
Resistin induces HCT-116 and SW-48 cell adhesion to HUVECs. HCT-116 and SW-48 cells were maintained as controls or stimulated with resistin and then stained with a crystal violet solution. The adhesion of HCT-116 and SW-48 cells to HUVECs was assessed. HCT-116 and SW-48 cells incubated with resistin (50 ng/mL) plus resistin-neutralizing antibodies (Ab) exhibited no increase in HUVEC adhesion. Data represent the mean ± standard error of the mean (SEM) from four independent experiments. * *p* < 0.05 *vs.* control cells; ^#^ *p* < 0.05 *vs.* cells treated with resistin only.

To determine the level of CRC cell adhesion to the endothelium, HCT-116 (p53-negative) and SW-48 (p53-positive) cells were maintained as controls or stimulated with resistin at 5, 10, 25 and 50 ng/mL for 4 h. After co-culturing both cell lines with HUVECs for 1 h and then removing non-adherent cells, the remaining adherent cells were stained with crystal violet solution and measured using a spectrophotometer. Treating cells with resistin increased the adhesion of both HCT-116 and SW-48 cells to HUVECs in a dose-dependent manner compared with untreated control (Figure 1). This effect was specific to resistin, as resistin-neutralizing antibodies completely inhibited the resistin-increased HCT-116 and SW-48 cell adhesion to HUVECs. We further investigated the molecular mechanism of this resistin effect by treating HCT-116 cells with 50 ng/mL of resistin.

### 2.2. Resistin Increases the Expressions of ICAM-1 and VCAM-1 in HCT-116 Cells

**Figure 2:**
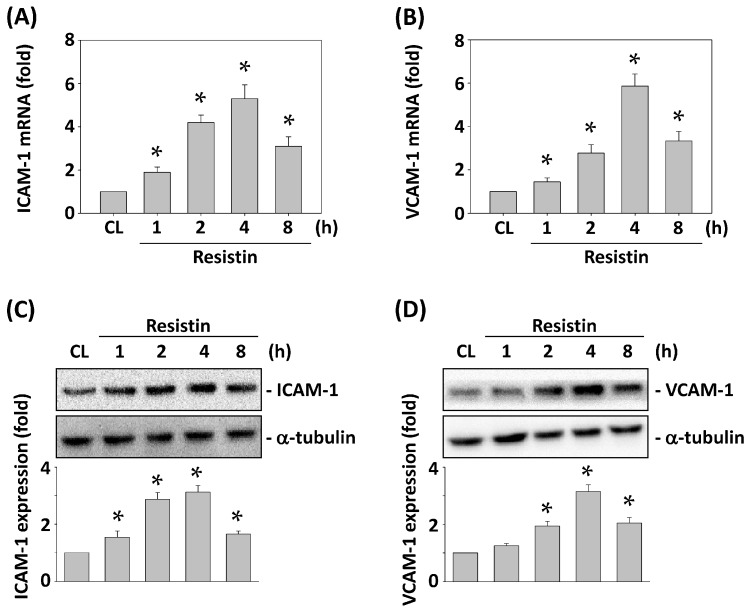
Resistin increases the expressions of ICAM-1 and VCAM-1 in HCT-116 cells. HCT-116 cells were maintained as control or stimulated with resistin. ICAM-1 and VCAM-1 mRNA (**A**,**B**) and protein (**C**,**D**) expression was analyzed. Data in (**A**,**B**) represent the mean ± SEM from three independent experiments. The results in (**C**,**D**) are representative of three independent experiments with similar results. * *p* < 0.05 *vs.* control cells.

Both ICAM-1 and VCAM-1 play a crucial role in cancer metastasis [15,16]. Hence, we determined whether resistin induces ICAM-1 and VCAM-1 expression in HCT-116 cells. HCT-116 cells were maintained as control or stimulated with resistin for 1, 2, 4 and 8 h, and the mRNA and protein expression of ICAM-1 and VCAM-1 were analyzed. Treating cells with resistin significantly increased ICAM-1 and VCAM-1 mRNA (Figure 2A,B) and protein (Figure 2C,D) expression within 1 h compared with the untreated control. The increased levels reached a maximum within 4 h and then declined but remained elevated after 8 h of treatment.

### 2.3. Blocking ICAM-1 and VCAM-1 in HCT-116 Cells Inhibits Adhesion to HUVECs

**Figure 3:**
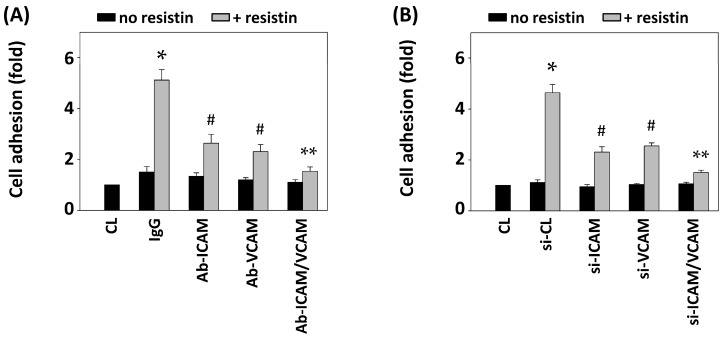
Blocking ICAM-1 and VCAM-1 in HCT-116 cells inhibits their adhesion to HUVECs. HCT-116 cells were pretreated with specific neutralizing antibodies (**A**) or siRNAs (**B**) for control (IgG/si-CL), ICAM-1, VCAM-1, or both and then were maintained as control or stimulated with resistin. HCT-116 cell adhesion was determined. Data represent the mean ± SEM from four independent experiments. * *p* < 0.05 *vs.* control cells (CL); ^#^ *p* < 0.05 *vs.* cells pretreated with IgG or si-CL and then treated with resistin only; ** *p* < 0.05 *vs.* cells pretreated with ICAM-1 or VCAM-1 neutralizing antibody and then treated with resistin.

To determine whether the induction of both ICAM-1 and VCAM-1 expression under resistin stimulation in HCT-116 cells regulates the HUVEC adhesion of HCT-116 cells, HCT-116 cells were pretreated with ICAM-1 and VCAM-1 specific blocking antibodies or siRNAs and then were maintained as control or stimulated with resistin for 4 h. The resistin-increased HUVEC adhesion of HCT-116 cells was inhibited by ICAM-1 or VCAM-1 antibody pretreatment. Co-pretreatment with both antibodies resulted in greater inhibitory effects compared to IgG and single-antibody pretreated cells (Figure 3A). Moreover, these inhibitory effects on the resistin-increased HUVEC adhesion of HCT-116 cells were further verified by transfecting HCT-116 cells with ICAM-1- and/or VCAM-1-specific siRNAs, which showed similar results to the blocking antibody pretreatment (Figure 3B).

### 2.4. Resistin-Initiated HCT-116 Adhesion to HUVECs Is Mediated by the NF-κB Activation

**Figure 4:**
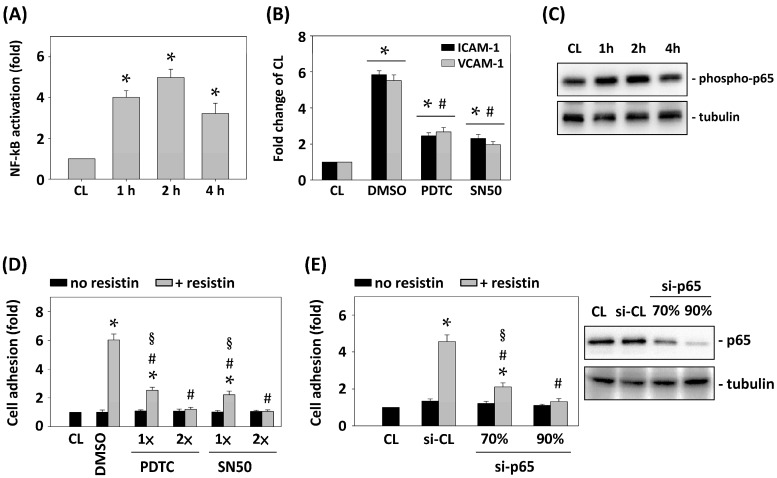
Resistin-initiated HCT-116 adhesion to HUVECs is mediated by NF-κB activation. (**A**) and (**C**) HCT-116 cells were maintained as control or stimulated with resistin for 1, 2, and 4 h and then NF-κB activation (**A**) and p65 phosphorylation (**C**) were determined by ELISA kit and Western blot, respectively; (**B**,**D**,**E**) HCT-116 cells were pretreated with (**B**,**D**) DMSO or NF-κB inhibitors (PDTC or SN50) and (**E**) control or p65 specific siRNA and then stimulated with resistin. HCT-116 cell adhesion and ICAM-1/VCAM-1 mRNA expression were analyzed. Data represent the mean ± SEM from three independent experiments. * *p* < 0.05 *vs.* control cells; ^#^ *p* < 0.05 *vs.* DMSO or si-CL cells with resistin treatment; ^§^ *p* < 0.05 *vs.* PDTC, SN50, or si-p65 cells without resistin treatment.

ICAM-1 and VCAM-1 expression are mainly regulated by the transcription factor NF-κB [15,16]. Therefore, we further determined whether NF-κB activation in HCT-116 cells regulates resistin-initiated HCT-116 cell adhesion to HUVECs. HCT-116 cells were maintained as control or stimulated with resistin for 1, 2, and 4 h, and the NF-κB activity was analyzed using the NF-κB activation ELISA kit. Treatment with resistin for 1, 2, and 4 h induced NF-κB activation within 1 h, which reached a maximum level within 2 h and then declined after 4 h (Figure 4A). Pretreating HCT-116 cells with DMSO or the NF-κB inhibitors pyrrolidine dithiocarbamate (PDTC) (20 μM) or SN50 (50 μg/mL) for 1 h and then stimulating cells with resistin for 4 h revealed that pretreating cells with PDTC or SN50 significantly inhibited the resistin effects on the expression of ICAM-1 and VCAM-1 in HCT-116 cells (Figure 4B). We further demonstrated that resistin induced NF-κB p65 phosphorylation in HCT-116 cells (Figure 4C). Moreover, Pretreating HCT-116 cells with DMSO or different doses of NF-κB inhibitors, PDTC: 20 (1×) and 40 (2×) μM or SN50: 50 (1×) and 100 (2×) μg/mL, revealed the dose-dependent inhibitory effect of inhibitors on the adhesion of HCT-116 cells to HUVECs in response to resistin stimulation (Figure 4D). Gene knockdown of p65 by different doses of p65 specific siRNA (20 and 30 nM), which caused 70% and over 90% reduction separately in the expression of the corresponding proteins, significantly inhibited the adhesion of HCT-116 cells to HUVECs in a dose-dependent manner under resistin stimulation (Figure 4E).

### 2.5. FA (Fulvic Acid) Inhibits Resistin-Initiated HCT-116 Cell Adhesion to HUVECs

**Figure 5:**
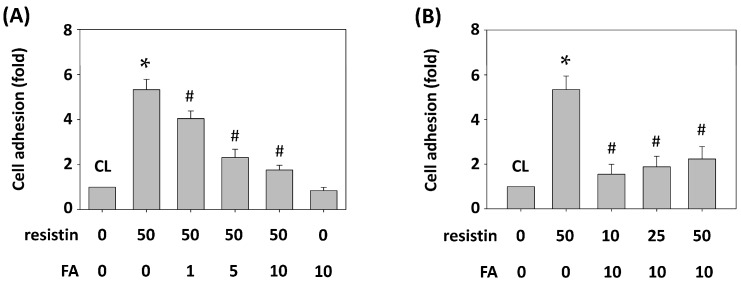
FA inhibits resistin-initiated HCT-116 cell adhesion to HUVECs. (**A**) HCT-116 cells were pretreated with FA at 0, 1, 5 and 10 μg/mL and then were maintained as control or stimulated with resistin; (**B**) HCT-116 cells were pretreated with FA at 0 or 10 μg/mL and then were maintained as control or stimulated with resistin at 10, 25 and 50 ng/mL. HCT-116 cell adhesion was determined. Data represent the mean ± SEM from three independent experiments. * *p* < 0.05 *vs.* control cells; ^#^ *p* < 0.05 *vs.* cells treated with resistin only.

FA is a potential therapeutic candidate for many diseases [1,4]. Hence, we determined whether the effects of resistin on the HUVEC adhesion of HCT-116 cells are modulated by FA. HCT-116 cells were pretreated with FA at 0, 1, 5 and 10 μg/mL for 1 h and then were maintained as control or stimulated with resistin for 4 h, and HCT-116 cell adhesion to HUVECs was examined. Treating cells with only resistin increased HCT-116 cell adhesion to HUVECs compared with the untreated control. However, pretreating cells with FA significantly inhibited the resistin effects on the HUVEC adhesion of HCT-116 cells in a dose-dependent manner (Figure 5A). Treating cells with FA (10 μg/mL) alone did not induce the adhesion of HCT-116 cells to HUVECs (Figure 5A). Moreover, pretreating HCT-116 cells with 0 or 10 μg/mL of FA for 1 h and then maintaining them as control or stimulating with resistin at 10, 25 and 50 ng/mL for 4 h showed that FA significantly inhibited the effects of all three concentrations of resistin on the HUVEC adhesion of HCT-116 cells compared with the resistin-only treated cells (Figure 5B).

### 2.6. FA Inhibits Resistin-Induced ICAM-1 and VCAM-1 Expression in HCT-116 Cells

**Figure 6:**
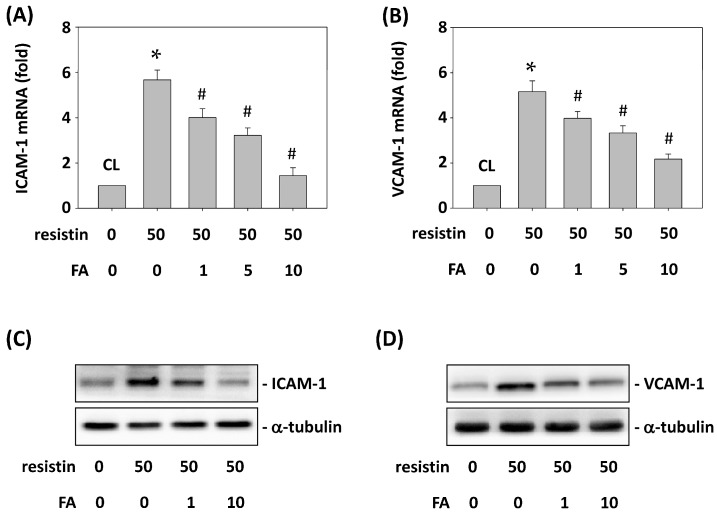
FA inhibits resistin-induced ICAM-1 and VCAM-1 expression in HCT-116 cells. HCT-116 cells were pretreated with FA at 0, 1, 5 and 10 μg/mL and then were maintained as control or stimulated with resistin. ICAM-1 and VCAM-1 mRNA (**A**,**B**) and protein (**C**,**D**) expression was determined. Data in (**A**,**B**) represent the mean ± SEM from three independent experiments. The results in (**C**,**D**) are representative of three independent experiments with similar results. * *p* < 0.05 *vs.* control cells; ^#^ *p* < 0.05 *vs.* cells treated with resistin only.

Next, we investigated whether FA mediates the resistin-increased ICAM-1 and VCAM-1 expression in HCT-116 cells. HCT-116 cells were pretreated with FA at 0, 1, 5 and 10 μg/mL for 1 h and then were maintained as control or stimulated with resistin for 4 h. The ICAM-1 and VCAM-1 mRNA and protein expression in HCT-116 cells was determined. Treating cells with only resistin induced ICAM-1 and VCAM-1 mRNA (Figure 6A,B) and protein (Figure 6C,D) expression compared with the untreated control. However, pretreating cells with FA at 1, 5 and 10 μg/mL significantly decreased ICAM-1 and VCAM-1 mRNA and protein expression in HCT-116 cells compared with the resistin-only treated cells.

### 2.7. FA Inhibits the Resistin Effect on HCT-116 Cell Adhesion to HUVECs by Down-Regulating NF-κB Activity

**Figure 7:**
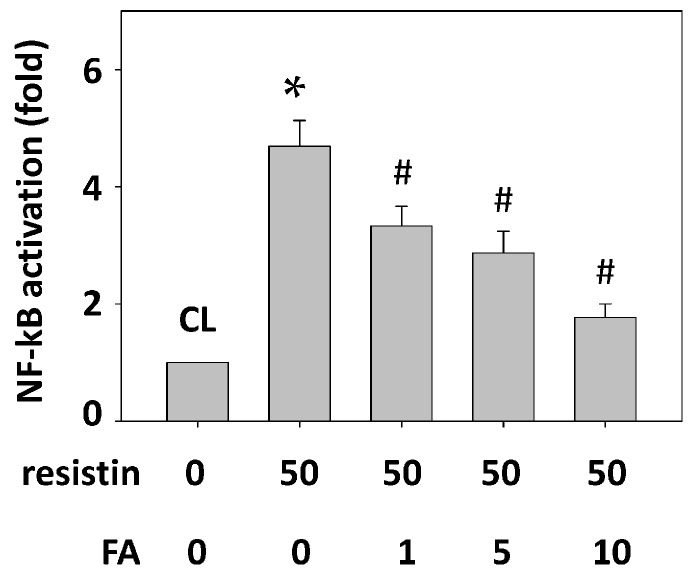
FA inhibits the resistin effect on HCT-116 cell adhesion to HUVECs by down-regulating NF-κB activity. HCT-116 cells were pretreated with FA at 0, 1, 5 and 10 μg/mL and then were maintained as control or stimulated with resistin. The activation of NF-κB was determined. Data represent the mean ± SEM from three independent experiments. * *p* < 0.05 *vs.* control cells; ^#^ *p* < 0.05 *vs.* cells treated with resistin only.

HCT-116 cells were pretreated with FA at 0, 1, 5 and 10 μg/mL for 1 h and then were maintained as control or stimulated with resistin for 4 h, and NF-κB activity was determined. Treatment with resistin alone activated NF-κB in HCT-116 cells (Figure 7, compared with the untreated control). However, pretreatment with FA at 1, 5 and 10 μg/mL significantly decreased NF-κB activity in HCT-116 cells.

## 3. Discussion

This study has made the unique discovery that resistin can initiate the endothelial adhesion of CRC cells through increasing NF-κB activity and the consequent ICAM-1 and VCAM-1 expression in HCT-116 cells. FA may play an antagonistic role to suppress this resistin effect. Our study has generated the following findings. First, this work shows that resistin increases both HCT-116 (p53-negative) and SW-48 (p53-positive) cell adhesion to HUVECs and demonstrated that this resistin effect is p53 tumor suppressor-independent. Second, the resistin-increased HCT-116 cell adhesion to HUVECs occurs through the induction of ICAM-1 and VCAM-1 mRNA and protein expression by the canonical NF-κB pathway, p65 subunit phosphorylation, in HCT-116 cells. Finally, FA attenuates the resistin effect on HCT-116 cell adhesion to HUVECs by inhibiting NF-κB activity and ICAM-1 and VCAM-1 expression. Thus, these results elucidate the molecular basis of resistin in promoting the adhesion of CRC cells to endothelium and the antagonistic role of FA in this effect.

Natural organic substrates are important and interesting sources in developing healthy foods and drugs. Recently, FA, derived from the humification of natural organic substrates, has been widely considered an attractive pharmacological research candidate because of its structure and composition of multiple active groups [3]. For example, the anti-oxidative activity [4] and anti-microbial [5], anti-allergy [23], anti-inflammation [6], and ultraviolet (UV)-protective properties [24] of FA have been elucidated and demonstrated. Moreover, the latest report indicated that FA interacts with human serum transferring, and this interaction might be applied to drug delivery design and tumor treatment [1]. Our present study investigates the effect of FA on the endothelial adhesion of cancer cells and demonstrates for the first time that FA exerts inhibitory effects on resistin-induced HCT-116 CRC cell adhesion to HUVECs, thereby possibly serving as potent inhibitory candidate in the endothelium adhesion of cancer cells during metastasis. Our results also prove that one mechanism of this FA effect is through inhibiting resistin-activated NF-κB in HCT-116 cells. This mechanism is similar to the reported anti-inflammatory activity of FA, which decreases TNF-α release in U937 monocyte-like cells and NF-κB activation in HUVECs [24]. Recently, cancer has also been identified as a chronic inflammatory disease [16,25]. Therefore, we suggest that FA may be applied in studying cancer treatment through blocking the inflammatory reactions in the cancer microenvironment, including monocyte, cancer, and endothelial cells.

Increasing epidemiological evidence since 2012 has demonstrated that the serum resistin level positively correlates to CRC carcinogenesis [26]. However, to our knowledge, the present study is the first to elucidate that part of the mechanism whereby resistin positively enhances CRC cell adhesion to the endothelium is p53 tumor suppressor-independent and occurs through inducing canonical NF-κB activity to up-regulate the genes encoding the ICAM-1 and VCAM-1 adhesion proteins in HCT-116 cells. Previously studies have demonstrated that NF-κB p65 subunit and p53 regulate each other’s activity to affect target gene transcription and cell function [27]. However, we excluded this possibility in our model, as resistin could induce the adhesion of p53-negative HCT-116 and p53-positive SW-48 cells to HUVECs. Resistin has been reported to exert its biological effects by binding to Toll-like receptor 4 (TLR4) on the surface of target cells [28]. NF-κB is an important transcription factor that can be activated by TLR4 which leads to potentiate expression of various genes involved in inflammation [29]. Prior published work has indicated that the canonical NF-κB is an important positive regulator of ICAM-1 and VCAM-1 gene transcription [15,30], and also enhanced monocyte adhesion to HUVECs [21]. Here, by employing several NF-κB-specific inhibitors, we evaluated the functional role of the canonical NF-κB pathway in the mechanism whereby resistin enhances CRC cell adhesion. Treatment of CRC cells with high doses of PDTC, SN50, or p65 siRNA efficiently blocked resistin-mediated CRC cell adhesion to HUVECs. Thus, our results demonstrate that resistin-induced NF-κB activation promotes CRC cell adhesion, in conjunction with inducing their ICAM-1 and VCAM-1 expression. Moreover, our results suggest that high serum resistin levels are related to cancer-associated chronic inflammation and support the previous study in which resistin exhibited pro-inflammatory properties by up-regulating the expression of TNF-α and IL-6 and the activation of monocytes [25,26,27,31,32,33]. Although resistin is an adipose tissue-secreted cytokines, recent studies have found that resistin could also be expressed in peripheral blood mononuclear cells, macrophages, and bone marrow cells [25,34,35]. Some reports have also indicated that the resistin releases the modulatory activities in inflammatory related diseases, including CRC [36,37,38,39,40]. Moreover, S. Ghaemmaghami *et al.* [25], indicated that resistin is not expressed in CRC cells and may act in a paracrine manner for CRC carcinogenesis. Inflammatory cells, including monocytes, but not CRC cells could play a major role in the high resistin expression in tissue and plasma levels due to the chronic low-grade inflammatory status in CRC. Although there is still some controversy over the precise role of high serum resistin and CRC cancer development, our study provides evidence that resistin modulates CRC cell adhesion to the endothelium.

Cancer metastasis, a major clinical problem of human cancer, is responsible for most cancer patient mortality [16]. The endothelial adhesion of cancer cells plays a crucial role in cancer metastasis. This is an important step for executing the subsequent steps of invasion and survival of cancer cells [41,42]. In the present study, we demonstrated that the resistin-initiated HCT-116 cell adhesion to the endothelium is achieved through inducing the expression of ICAM-1 and VCAM-1 in HCT-116 cells. ICAM-1 and VCAM-1 are two important cell surface adhesion molecules that are mainly expressed in endothelial cells to modulate the immune process and cancer cell adhesion to the endothelium [16,42]. Resistin increases ICAM-1 and VCAM-1 expression in endothelial cells to induce monocyte-endothelial cell adhesion [21]. However, the present study shows that resistin not only induces ICAM-1 and VCAM-1 expression in CRC cells but also mediates cancer cell adhesion to the endothelium. Recently, soluble forms of adhesion molecules, including ICAM-1 and VCAM-1, have been found in the serum of cancer patients, in the supernatant of cytokine-activated endothelial cells and in the culture medium of CRC cell lines [43,44]. Moreover, an elevated serum-soluble adhesion molecule level correlates significantly with cancer progression and can serve as clinical diagnostic and prognostic markers [15]. Therefore, although we have not yet determined the precise role of ICAM-1 and VCAM-1 expression in CRC cells, ICAM-1 and VCAM-1 in the resistin-treated cancer and endothelial cells may be secreted into the cancer microenvironment and mediate the interactions of cancer, immune, and endothelial cells. On the other hand, recent studies have indicated that cancer metastasis could be initiated by cancer cell-leukocyte interactions [45]. The specific receptors of ICAM-1 and VCAM-1, *i.e.*, lymphocyte function-associated antigen (LFA)-1 and very-late antigen (VLA)-4 integrins, respectively, are expressed in immune cells [46]. Thus, the issue of whether the resistin-initiated endothelial adhesion of CRC cells through ICAM-1 and VCAM-1 in CRC cells is controlled by cancer cell-leukocyte interactions warrants further investigation.

This study has indicated that resistin can initiate the endothelial adhesion of CRC cells, with concomitant increases in NF-κB activity and ICAM-1 and VCAM-1 expression in CRC cells. However, FA antagonizes this resistin-induced response. As a result, this study proposes novel roles for resistin and FA in modulating CRC adhesion to the endothelium. Moreover, a new perspective on the antagonistic effects of FA on the endothelial adhesion of cancer cells warrants further exploration.

## 4. Experimental Section

### 4.1. Materials

Recombinant human resistin was purchased from R & D Systems (Minneapolis, MN, USA). FA was supplied from the Esther Material Technology Co., Ltd., Kaohsiung, Taiwan. Human polyclonal antibodies against p65, ICAM-1 and VCAM-1 were purchased from R & D Systems (Minneapolis, MN, USA). Mouse monoclonal anti-resistin neutralizing antibody was purchased from Biocompare. The specific siRNAs for control, ICAM-1, VCAM-1, and p65 were purchased from Invitrogen (Carlsbad, CA, USA). NF-κB inhibitors (PDTC and SN50) and all other chemicals of reagent grade were purchased from Sigma (St. Louis, MO, USA) unless otherwise noted.

### 4.2. Cell Cultures

Human HCT-116 and SW-48 cells were obtained from the cell bank in the Food Industry Research and Development Institute (Hsinchu, Taiwan) and were grown in DMEM (Life Technologies, Inc., Gaithersburg, MD, USA) supplemented with 10% fetal bovine serum (FBS; Life Technologies, Inc., Gaithersburg, MD, USA) and 1% penicillin/streptomycin in a 37 °C incubator. Human umbilical vein endothelial cells (HUVECs) were obtained from Cambrex Bio Science (Walkersville, MD, USA) and were grown in endothelial cell growth medium-2 (EGM-2) and maintained in a 37 °C incubator. HUVECs of passage 3–5 were used for the experiments.

### 4.3. Cell Adhesion Assay

The binding of HCT-116 cells to HUVECs was determined using a colorimetric method [47]. Briefly, HCT-116 cells were grown in 0.5% FBS medium overnight and then were stimulated with resistin. For inhibition experiments, HCT-116 cells were pretreated with specific inhibitors or neutralizing antibodies or were transfected with specific siRNAs before stimulating with resistin. The HCT-116 cells (2 × 10^5^ cells/mL) were co-cultured with HUVECs, and non-adherent cells were removed by washing with PBS. The cells were fixed with 1.0% glutaraldehyde for 20 min and then stained with 0.1% crystal violet for 15 min. After washing with sterile water, the stained cells were solubilized in 0.1% Triton X-100 overnight. The adhesion level of HCT-116 cells were determined at 595 nm wavelength by using a spectrophotometer. The data were normalized by the background determined from the staining of HUVECs alone.

### 4.4. Western Blot Analysis

The cells were collected and lysed with RIPA buffer (1% nonylphenoxypolyethoxyethanol (NP)-40, 0.5% sodium deoxycholate, 0.1% SDS, and protease/phosphatase inhibitor cocktail (phenylmethylsulfonyl fluoride, aprotinin, and sodium orthovanadate)). The concentration of total cell lysate was determined using the protein assay kit (Bio-Rad, Hercules, CA, USA), separated by sodium dodecyl sulfate-polyacrylamide gel electrophoresis (SDS-PAGE) (10% running and 4% stacking), transferred onto a nitrocellulose membrane, and analyzed using the designated antibodies. Immunodetection was performed using the Western-Light chemiluminescent detection system (Applied Biosystems, Forster City, CA, USA).

### 4.5. Quantitative Real-Time PCR

RNA was extracted and converted to cDNA. The cDNA was amplified using the ABI Prism 7900HT with the FastStart DNA SYBR Green I kit (Invitrogen, Carlsbad, CA, USA) with primers for ICAM-1 (forward: 5′-GTGACATGCAGCACCTCCTG-3′; reverse: 5′-TCCATGGTGATCTCTCCTCA-3′), VCAM-1 (forward: 5′-CCGGATTGCTGCTCAGATTGGA-3′; reverse: 5′-AGCGTGGAATTGGTCCCCTCA-3′), and 18S rRNA (forward: 5′-CGGCGACGACCCATTCGAAC-3′; reverse: 5′-GAATCGAACCCTGATTCCCCGTC-3′). The quantification of gene expression levels was performed using the 2^−ΔΔ*C*t^ method. The PCR for all genes was performed in duplicate.

### 4.6. siRNA Transfection

HCT-116 cells at 70% confluence were transfected with the specific siRNA, which caused ~60%–90% reductions in the expressions of the corresponding proteins, for 48 h using the RNAiMAX transfection kit (Invitrogen, Carlsbad, CA, USA).

### 4.7. NF-κB Activation Enzyme-Linked Immunosorbent Assay (ELISA)

The cell nuclear extracts were purified using a nuclear protein extract kit (EK1121) (Panomics, Redwood City, CA, USA), and the activation of NF-κB p65 in equal amounts of nuclear proteins was measured using commercially available ELISA kits (EK1121) (Panomics, Redwood City, CA, USA). Briefly, an oligonucleotide containing a NF-κB 65 consensus binding site (supplied by the ELISA kit) was immobilized on the 96-well plate. Nuclear proteins were added to the plate to bind to this oligonucleotide. The p65/DNA complex was identified by a NF-κB p65 specific antibody and a secondary horseradish peroxidase (HRP)-conjugated antibody and was determined by using spectrophotometry.

### 4.8. Statistical Analysis

The experiment was performed at least three times. Results are represented as the mean ± standard error of mean (SEM). Statistical significance was calculated by using an independent Student’s paired *t* test for comparisons of two groups of data and an ANOVA followed by Scheffe’s test for multiple comparisons. *p* value less than 0.05 was considered to be significant.
